# A Rapid HPLC-UV Protocol Coupled to Chemometric Analysis for the Determination of the Major Phenolic Constituents and Tocopherol Content in Almonds and the Discrimination of the Geographical Origin

**DOI:** 10.3390/molecules26185433

**Published:** 2021-09-07

**Authors:** Natasa P. Kalogiouri, Petros D. Mitsikaris, Dimitris Klaoudatos, Athanasios N. Papadopoulos, Victoria F. Samanidou

**Affiliations:** 1 Laboratory of Analytical Chemistry, Department of Chemistry, Aristotle University of Thessaloniki, 54124 Thessaloniki, Greece; samanidu@chem.auth.gr; 2 Laboratory of Chemical Biology, Department of Nutritional Sciences and Dietetics, International Hellenic University, Sindos, 57400 Thessaloniki, Greece; petrosmitsikaris@gmail.com (P.D.M.); papadnas@ihu.gr (A.N.P.); 3 Laboratory of Oceanography, Department of Ichthyology and Aquatic Environment, School of Agricultural Sciences, University of Thessaly, 38446 Volos, Greece; dklaoud@uth.gr

**Keywords:** almonds, HPLC, authenticity, PCA, tocopherols, phenolics

## Abstract

Reversed phase-high-pressure liquid chromatographic methodologies equipped with UV detector (RP-HPLC-UV) were developed for the determination of phenolic compounds and tocopherols in almonds. Nineteen samples of *Texas* almonds originating from USA and Greece were analyzed and 7 phenolic acids, 7 flavonoids, and tocopherols (−α, −β + γ) were determined. The analytical methodologies were validated and presented excellent linearity (r^2^ > 0.99), high recoveries over the range between 83.1 (syringic acid) to 95.5% (ferulic acid) for within-day assay (*n* = 6), and between 90.2 (diosmin) to 103.4% (rosmarinic acid) for between-day assay (*n* = 3 × 3), for phenolic compounds, and between 95.1 and 100.4% for within-day assay (*n* = 6), and between 93.2–96.2% for between-day assay (*n* = 3 × 3) for tocopherols. The analytes were further quantified, and the results were analyzed by principal component analysis (PCA), and agglomerative hierarchical clustering (AHC) to investigate potential differences between the bioactive content of almonds and the geographical origin. A decision tree (DT) was developed for the prediction of the geographical origin of almonds proposing a characteristic marker with a concentration threshold, proving to be a promising and reliable tool for the guarantee of the authenticity of the almonds.

## 1. Introduction

The current trend in nutrition is following the Mediterranean diet, as it is considered one of the healthiest dietary patterns. Nuts are a highly nutritious food with unique taste and beneficial health properties deriving from their unique molecular composition. Popular tree nuts comprise almonds (*Prunus amygdalus Batsch* or *P. dulcis*), walnuts (*Juglans regia* L.), hazelnuts (*Corylus avellane* L.), and pistachios (*Pistachia vera* L.), among others. Almonds are one of the most popular and widely harvested culinary nuts in the world. Apart from their unique taste and texture, they have been proven to possess a wide variety of beneficial health properties deriving from their unique molecular composition. Thus, they are now considered as an important component of a healthy and highly nutritious diet [1,2,3,4,5].

Numerous studies have shown that various pharmacological activities can be attributed to regular consumption of almonds. A meta-analysis observed a significant reduction in LDL-C levels with almond consumption [6]. Additionally, a systematic review conducted by Kalita et al. [7] suggests that eating almonds leads to a significant reduction in total cholesterol, LDL-C, and triglycerides levels, whilst the impact on HDL-C levels is minor. In a randomized, controlled crossover study that took place over a time period of six weeks, individuals that consumed 45 g of almonds per day showed reduced LDL-C and non-HDL-C levels and, at the same time, maintained their HDL-C levels [8]. The study also demonstrated that almond intake reduced abdominal fat which is of very high significance, considering the fact that high amounts of abdominal fat are a major factor in metabolic syndrome. Furthermore, studies suggest that apart from reducing the risk of cardiovascular disease, almonds exhibit anti-inflammatory and anti-carcinogenic effects [9]. From these studies accrues the conclusion that almonds can be an effective diet tool in the process of trying to decrease an individual’s cholesterol levels, hence reducing his risk of coming across any type of cardiovascular disease.

Τhe beneficial health effects are mainly owed to their favorable phytochemical composition. Almonds are rich in bioactive constituents, mainly in phenolics and tocopherols. These compounds are defined as secondary plant metabolites and originate from carbohydrates through the shikimate and phenyl propanoid pathways [2,10]. Their chemical structure is characterized by one or more aromatic rings bearing at least one hydroxyl group [2]. Tocopherols are one of two subgroups that comprise vitamin E, with the other one being tocotrienols. Tocopherols are constituted by four derivatives: alpha, beta, gamma, and delta [11,12,13]. It is suggested that phenolic compounds that are found in almond skins act synergistically with vitamins C and E and protect the LDL particles from oxidation, resulting in the overall enhancement of the individual’s antioxidant capacity [14].

Although polyphenols and tocopherols are ubiquitous in nuts, and particularly in almonds, their content, distribution and bioavailability vary depending on genetics, location, plant structure, pre- and post-harvest factors and climate conditions [15,16,17]. In this context, the analysis of almonds’ phenolic content could provide useful information, making the evaluation process of different almond cultivars produced in different countries more accurate. The authentication process of various almond cultivars also contributes to the assessment of overall almond quality. However, traditional methods of doing so depend largely on environmental and production factors, making the differentiation between cultivars, geographical origin, and type of farming a difficult task to tackle [15,18,19,20,21,22]. Hence, the need arises to develop specific analytical methodologies and protocols that are applicable to a wide variety of nut types, with the end goal of differentiating them based on their phenolic content.

The determination of small bioactive molecules from food matrices involves the examination of several distinct aspects of the analytical methodology. Separation of phenolic compounds and tocopherols is mainly achieved with high pressure liquid chromatography (HPLC) coupled to UV [12,23,24], photodiode array (DAD) [25,26], or mass spectrometric (MS) detectors [27,28]. The most crucial step of the analytical methodology is sample preparation. Several laborious and time-consuming protocols have been proposed, suggesting the use of large volumes of organic solvents and Soxhlet-type apparatus [29,30]. The objective is to eliminate the use of organic solvents, minimize extraction times and select techniques that are suitable for the rapid determination of bioactive constituents [23]. The further processing of the results with chemometric tools increases the extensiveness of the analysis, enlightening the reliability of the conclusions derived from the experimental data. Data mining and the development of chemometric models are widely used in food authenticity studies for the investigation of several issues such as the discrimination of botanical origin, geographical origin, farming type, etc. [31,32,33].

The objective of this research was to develop two rapid HPLC-UV methodologies for the determination of the major phenolic compounds and tocopherols in almonds of the *Texas* variety originating from Greece and the USA. Τhe quantification results were further analyzed with agglomerative hierarchical clustering (AHC) and principal component analysis (PCA) to investigate similarities between samples of the same geographical origin. A decision tree (DT) was developed for the classification of almonds, proving to be a promising and reliable tool for verifying the geographical origin on the basis of their phenolic profile and bioactive content.

## 2. Results

### 2.1. Analytical Performance

#### 2.1.1. RP-HPLC-UV Method for the Determination of Phenolic Compounds

The analytical parameters of the HPLC-UV methodology for the determination of phenolic compounds, including the calibration curves, the linear range, the determination coefficients, the limits of detection (LODs) and limits of quantification (LOQs), precision and accuracy are summarized in Table S1. As it can be observed, the coefficients of determination ranged between 0.991 and 0.999, showing good linearity for all the phenolic analytes. The LOQs were found to range between 0.24 (rosmarinic acid) to 1.80 μg/g (diosmin), while the LODs were calculated equal to 0.08 (rosmarinic acid) to −0.60 μg/g (vanillin). The RSD% of the within-day (*n* = 6) and between-day assays (*n* = 3 × 3) was lower than 6.1 and 10.3, respectively, presenting adequate precision. The accuracy was assessed by means of relative percentage of recovery (%R) at three concentration levels (0.5, 5, 10 μg/g) and ranged between 83.1 (syringic acid at 10 μg/g concentration level) to 95.5% (ferulic acid at 0.5 μg/g concentration level) for within-day assay (*n* = 6) (Table S2), and between 90.2 (diosmin) to 103.4% (rosmarinic acid) for between-day assay (*n* = 3 × 3) (Table S3).

#### 2.1.2. RP-HPLC-UV for the Determination of Tocopherols

The analytical parameters of the RP-HPLC-UV methodology for the determination of tocopherols are presented in Table S4. The LOQs were found to range between 0.36 (γ-tocopherol) to −0.99 μg/g (α-tocopherol), while the LODs were calculated equal to 0.12 (γ-tocopherol) to −0.33 μg/g (α-tocopherol). The RSD% of the within-day (*n* = 6) and between-day assays (*n* = 3 × 3) was lower than 5.5 and 8.1, respectively, presenting adequate precision. The accuracy was assessed by means of relative percentage of recovery at three concentration levels (0.5, 5, 10 μg/g) and ranged between 95.1 and 100.4% for within-day assay (*n* = 6) (Table S5), and between 93.2–96.2% for between-day assay (*n* = 3 × 3) (Table S6).

### 2.2. Real Samples’ Application

#### 2.2.1. Determination of Phenolic Compounds

Nineteen almond samples of the *Texas* variety from USA and Greece were analyzed. In total, fourteen phenolic compounds were determined. Gallic acid, ferulic acid, sinapic acid, rosmarinic acid, vanillic acid, p-coumaric, and caffeic acid were determined from the class of phenolic acids. Diosmin, catechin, epicatechin, quercetin, luteolin, apigenin, and kaempferol were determined from the class of flavonoids. A characteristic chromatogram, of a spiked sample at 5 μg/g is presented in the Supplementary Materials Figure S1. The retention times of the identified phenolic analytes are presented in Table S7. All samples were analyzed in triplicate and the concentration ranges as well as the mean values (±SD) are presented in Table 1.

The results are in accordance with Coric et al. [26] and Boiling [34]. Specifically, vanillic acid ranged between 1.37 to 4.25 μg/g in Greek almonds and between 1.03 to 2.23 μg/g in American almonds, similarly to Coric et al. [26] who reported a range of 0.38–2.84 μg/g. Caffeic acid ranged between 1.18 to 1.85 μg/g in Greek almonds and between 0.82 to 1.90 μg/g in American almonds, slightly higher concentrations compared to Coric et al. [26] who reported concentrations up to 1.48 μg/g. Sinapic acid ranged between 1.25 to 4.48 μg/g in Greek almonds, and between 1.02 to 3.65 μg/g in American almonds, correspondingly to Coric et al. [26] who reported concentrations up to 3.50 μg/g. Syringic acid was not detected in any of the samples, while p-coumaric acid was detected below the LOQ. Furthermore, rosmarinic acid ranged between 1.03 to 1.84 μg/g in Greek almonds and between 2.51 to 4.19 μg/g in American almonds. The detected concentrations of rosmarinic acid are higher than those reported previously by Keser et al. [35]. Significantly high concentrations up to 4.56 μg/g in Greek almonds and up to 1.81 μg/g in American almonds were detected for gallic acid, as well, compared to the literature [26,34,36].

As far as flavonoids are concerned, catechin was the dominant phenolic compound with similar mean values of 21.3 μg/g for Greek and 20.2 μg/g for American, respectively. The second most abundant flavonoid was diosmin with a higher mean value of 8.06 μg/g in American almonds, compared to Greek almonds (3.91 μg/g). Higher concentrations of apigenin were detected in Greek almonds over the range 4.65 to 8.65 μg/g compared to American almonds (up to 3.21 μg/g). The mean concentration of luteolin in Greek almonds was found equal to 0.59 μg/g, while it was not detected in American almonds. Epicatechin ranged between 3.21–6.01 μg/g in Greek almonds and between 1.02 to 1.21 μg/g in American almonds. Kaempferol was detected in Greek almonds at a higher concentration with a mean value of 2.53 μg/g compared to 1.30 μg/g that was detected in American almonds, similarly to Coric et al. [26] who reported concentrations up to 2.63 μg/g. Finally, quercetin was detected at a mean concentration of 0.53 μg/g in Greek almonds and was not detected in American almonds, since according to the literature [28,34], the glucoside is mainly dominant in almonds and not its aglycone form.

#### 2.2.2. Determination of Tocopherols

The separation of tocopherols was achieved within 15 min. The gradient elution program performed separation of α-tocopherol at (Rt = 9.2 min) and δ-tocopherol (Rt = 12.1 min), while β + γ tocopherols co-eluted (Rt = 10.6 min) and were analyzed as a sum according to Gliszczyńska-Świgło et al. [9]. A representative chromatogram of the 10 μg/g standard solution mixture is shown in the Supplementary Materials Figure S2. The analysis of tocopherols proved that almonds constitute a great source of α-tocopherol which ranged between 502 to 802 μg/g and between 221 to 326 μg/g in American almonds. γ-Tocopherol was measured as the sum of β- and γ-tocopherol, since these tocopherols are isomers and co-elute in RP chromatographic systems. δ-Tocopherol was not detected in any of the analyzed samples. All samples were analyzed in triplicate and quantification ranges and the mean values (±SD) are presented in Table 2.

### 2.3. Chemometric Analysis

The quantification results of the determined phenolic compounds and tocopherols (Section 2.2.1 and Section 2.2.2) were further processed with chemometric tools to examine if the samples can be classified according to their phenolic composition and tocopherol content.

#### 2.3.1. PCA

**Figure 1:**
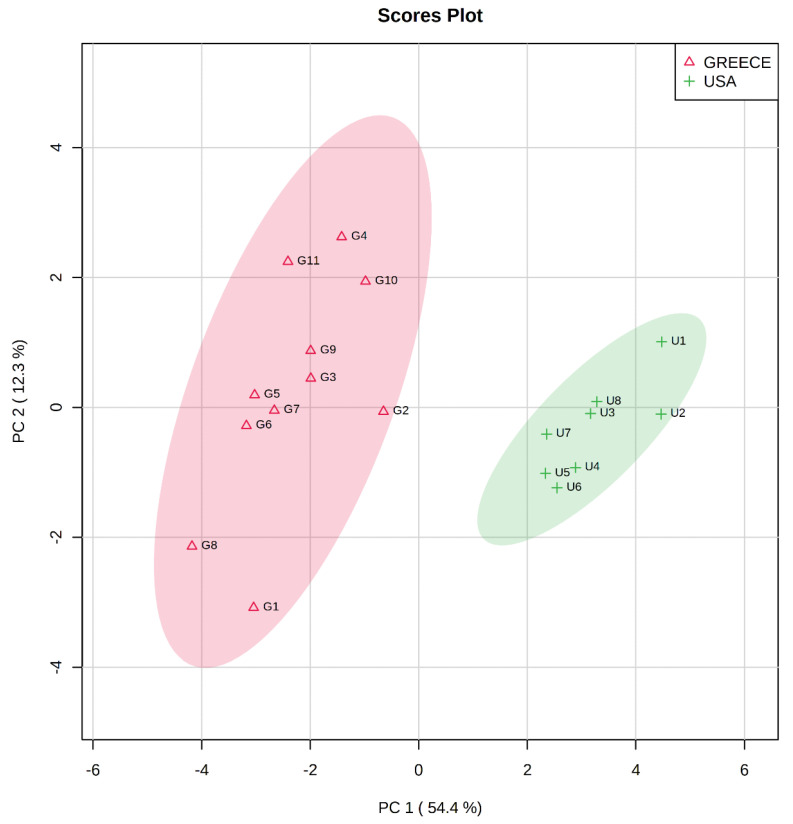
PCA Score plot showing the pairwise correlation between PCs in the clustering between Greek and American almonds.

PCA was applied in the analysis of nineteen different samples of almonds originating from Greece and the USA. The data matrix consisted of sixteen features (quantification results of phenolics and tocopherols) and was normalized using the auto-scaling function of the MetaboAnalyst package [37]. Figure 1 presents the score plot and the clustering of the almonds into two individual groups, according to the geographical origin. Almonds originating from Greece are marked in red and almonds originating from the USA are marked in green. The first two Principal Components (PCs) explained 66.8% of the variance, presenting appropriate groups of samples of the same variety and geographical origin. The PCA biplot in Figure S3 presents the influence of the variables in each PC.

#### 2.3.2. Agglomerative Hierarchical Clustering

Cluster analysis was employed to divide the matrix into homogeneous groups measuring the distance between each pair of objects and without previous knowledge about the structure of the groups. A tree diagram was built with AHC to identify the groups that present high similarity. Each object is considered a singleton cluster (leaf) by the algorithm. Subsequently, the pairs of clusters are merged until all of them end up into a large cluster that contains all the objects [38], resulting in a tree-based representation, the so-called dendrogram.

**Figure 2:**
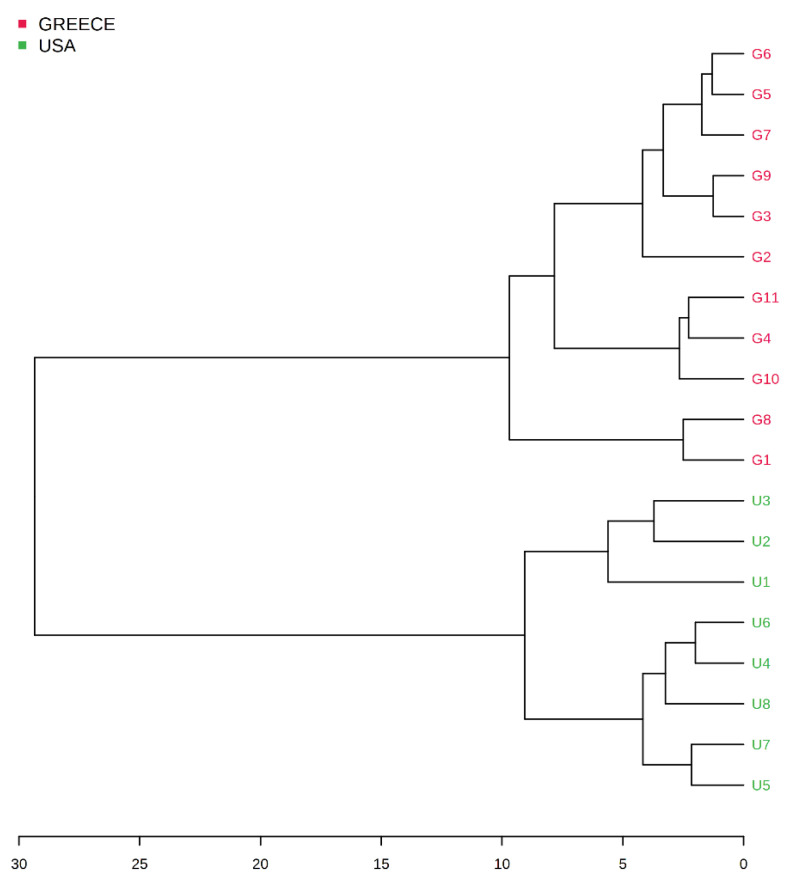
HCA dendrogram of Greek (G1–G11) and American (U1–U8) almonds’ clustering in two major groups.

Figure 2 presents the dendrogram of the eleven Greek and eight American almonds’ clustering in two major groups according to the place of origin.

**Figure 3:**
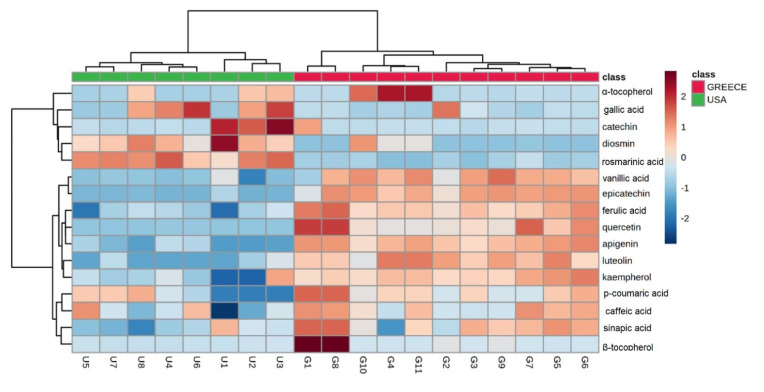
Clustered image map acquired by HCA dendrogram showing pairwise correlation between almonds produced in USA (U1–U8, in green color on the left-hand side of the heatmap) and Greece (G1–G11, in red color on the right-hand of the heatmap). The red blocks of the heatmap indicate positive correlations, whereas the blue blocks indicate negative correlations for the clustering of the samples. The lighter shades indicate smaller correlation values.

The heatmap in Figure 3 presents the data matrix showing pairwise correlations between the Greek (G1–G11) and American almonds (U1–U8). Each one of the colored cells corresponds to a concentration value; the samples are represented in the columns and the compounds in the rows.

#### 2.3.3. Decision Tree

The DT algorithm was built to develop a prediction model by splitting the data repeatedly into two discrete subsets according to the numerical value (i.e., concentration threshold) of the selected explanatory variable. The model selects the most significant variable that minimizes the model’s total error. The initial dataset was split into a training and a test set. Twelve samples were used as training set and seven as test set. The developed DT suggested that ferulic acid could be used as a characteristic marker for the discrimination between Greek and American almonds and succeeded in classifying the samples with zero error, resulting in two terminal nodes and setting the concentration threshold of 1.54 μg/g. The developed DT was validated with a receiver operating characteristics (ROC) plot for each class of almonds with 1-specificity and zero error (Figure S4).

**Figure 4:**
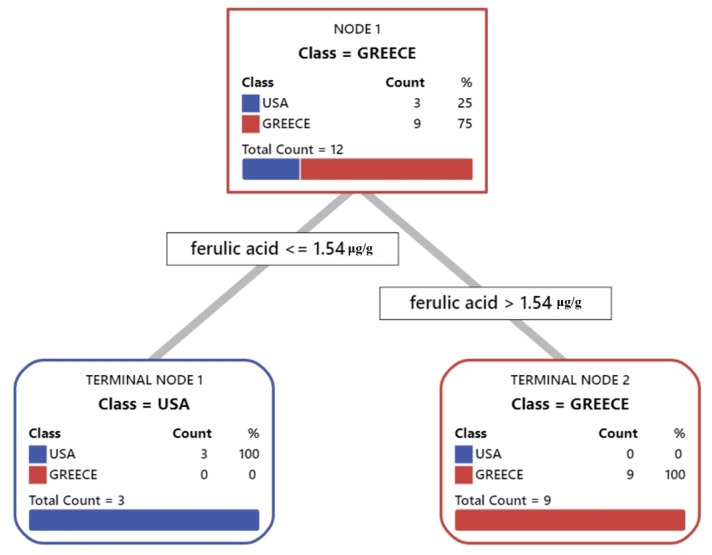
Optimal decision tree diagram using predictive analysis for phenolic and tocopherol concentration of almond samples originating from Greece and USA.

According to Figure 4, almonds with calculated concentrations lower or equal to 1.54 μg/g were produced in the USA, while those with higher concentrations than 1.54 μg/g were produced in Greece.

## 3. Materials and Methods

### 3.1. Chemicals and Standards

Acetonitrile (ACN) and 2-propanol (IPA), HPLC grade, were purchased by Panreac —AppliChem (Darmstadt, Germany). Methanol (MeOH), HPLC grade, was acquired by Carl Roth (Carlsruhe, Germany). Hexane reagent grade 99% and acetic acid 99% were purchased by Sigma-Aldrich (Steinheim, Germany). Ultrapure water was provided by a Milli-Q purification system (Millipore, Bedford, MA, USA).

Sinapic acid 95%, gallic acid 98%, ferulic acid 98%, rosmarinic acid 98%, catechin 98%, epicatechin 97%, p-coumaric 98%, quercetin 98%, diosmin 97%, kaempferol 97%, vanillic acid 97%, and caffeic acid 98% were purchased from Sigma-Aldrich (Steinheim, Germany). Luteolin 98% was acquired from Santa Cruz Biotechnologies. Apigenin 97% was purchased from Alfa Aesar (Karlsruhe, Germany). α-Tocopherol 96%, β-tocopherol 96%, γ-tocopherol 96%, and δ-tocopherol 96% were purchased by Sigma-Aldrich (Steinheim, Germany). Stock standard solutions of each analyte (1000 mg/L) were solubilized in methanol and stored at −20 °C in dark brown glass bottles.

### 3.2. Collection of Samples

Εleven Greek almond samples belonging to the variety *Texas* were acquired from different producers, originating from different territories around Greece (Evia, Trikala, Vergina, Katerini, Adendro, Elassona, Mouzaki, Aridaia, Veroia, Drama, Larissa), and eight almond samples of the *Texas* variety originating from California and available in the Greek market were acquired from eight different traders.

### 3.3. Instrumentation

The chromatographic analysis of the analytes was performed in an Agilent (Santa Clara, CA, USA) 1220 Infinity HPLC-UV, using gradient elution methods. The HPLC system consisted of the following: manual injector, column oven, degasser, and lastly, a UV Detector. In order to monitor the analysis, the Agilent Open Lab software and the package Method and Run Control were used. For data processing, the Data Analysis software package was used to identify and integrate the peaks. A glass vacuum-filtration apparatus, produced by Alltech Associates (Deerfeld, IL, USA), in combination with cellulose nitrate 0.22 μm nylon filters (Whatman Laboratory Division, Maidstone, UK) were utilized for the filtration of the aqueous and organic phase, respectively. QMax RR syringe filters (0.22 μm nylon membrane) were purchased from Frisenette ApS (Knebel, Denmark) and used for filtering the real samples prior to analysis. An ultrasonic bath (MRC: DC-150H) by MRC (Essex, UK) was utilized to remove the template from the MIP as well as for sample preparation. A vortex mixer from VELP Scientifica (Usmate Velate, Italy) was used for the agitation of the samples. A centrifuge system 3–16PK by Sigma (Osterode am Harz, Germany) was operated for centrifugation.

### 3.4. Chromatographic Conditions

A Nucleosil RP-18 analytical column (250 mm × 4.6 mm, 5 μm particle size), supplied by Macherey–Nagel (Düren, Nordrhein-Westfalen, Germany) was used for the analysis of polyphenols at 280 nm. The mobile phase consisted of a mixture of acetic acid in ultrapure water 2% *v/v* (A) and acetic acid in ACN 0.5% *v/v* (B). The system operated in a gradient mode at 28 °C. At the beginning of the analysis, the mixture was 100% A, gradually dropping to 20% A at the 60 min mark. The flow rate was set at 1 mL/min. Each chromatographic run lasted for 60 min. The peaks were identified by comparing the retention time of the standard compound with the peaks detected in real samples.

A Kromasil RP-18 analytical column (125 mm × 4.6 mm, 5 μm particle size), purchased from Macherey–Nagel, was used for the analysis of tocopherols at 295 nm. The mobile phase consisted of a mixture of methanol (A) and ACN (B). The system operated in a gradient mode at 28 °C. At the beginning of the analysis, the mixture was stable at 50% A and at the 7 min mark, it gradually increased to 100% A until the 12 min mark. The flow rate was set at 1 mL/min. Each chromatographic run lasted for 15 min. The peaks were identified by comparing the retention time of the standard compound with the peaks detected in real samples.

### 3.5. Sample Preparation

For the extraction of the phenolic compounds, a modified extraction protocol was applied, as previously suggested by Kritikou et al. [39]. In brief, 0.5 g of each homogenized sample was weighted in 2 mL Eppendorf tubes and 1 mL of MeoH: H_2_O (80:20, *v*/*v*) was added. The samples were vortexed for 1 min and then they were centrifuged for 5 min at 8000 rpm. The extract was collected and filtered through 0.45 μm nylon filters and 20 μL were injected in the chromatographic system. As for the extraction of tocopherols, 1 g of homogenized samples were weighted in 15 mL falcon tubes and 10 mL of hexane were added to extract the lipid fraction. The samples were vortexed for 1 min and they were then placed in an ultra-sound bath at 40 °C for 10 min. In a further step, the falcon tubes were centrifuged at 8000 rpm for 10 min. The organic layer was transferred and evaporated in a rotary evaporator under vacuum. The almond oil product was collected and stored in dark brown vials at −20 °C. Prior to analysis, 20 mg of oil was weighed and dissolved in 500 µL 2-propanol, according to Martakos et al. [40]. The mixture was filtered through nylon 0.45 µm syringe filters and an aliquot of 20 µL was injected into the HPLC system.

### 3.6. Method Validation

Μethod validation was performed for both methodologies to estimate selectivity, linearity, LODs and limits of quantification (LOQs), within-day, and between-day accuracy and precision, respectively. Linearity studies were performed in triplicate and covered the working range of 0.5–20 μg/g which was selected for the phenolic compounds, and the working range of 5–50 μg/g was selected for tocopherols. Linearity was assessed by constructing calibration curves for each analyte using standard solutions. Eight point calibration curves were constructed by plotting the peak area versus concentration. LODs and LOQs were calculated on the basis of the S/N of the analyte until an S/N ratio of 3:1 (LOD) and 10:1 (LOQ) was reached. [41].

Accuracy and precision were studied for both methods using a pool sample spiked at three different concentrations: 0.5 μg/g—10 μg/g—20 μg/g for phenolics and, 5 μg/g—25 μg/g—50 μg/g for tocopherols, all analyzed in triplicate. Relative recoveries (R%) were calculated by means of recovery percentage, by comparing the found and added concentrations of the examined analytes (mean concentration found/concentration*100, R%), expressing accuracy. The precision of the method was expressed in terms of relative standard deviation (RSD%). Following this approach, within-day precision (repeatability) was assessed in six replicates (*n* = 6), while between-day precision (reproducibility) were assessed by performing triplicate analysis for spiked samples within three consecutive days (*n* = 3 × 3) [41]. Five blank matrices were used to assess selectivity and no interferences were observed in the same chromatographic window for both methodologies.

### 3.7. Chemometric Analysis

PCA was used as a mathematical tool to represent the variation in the dataset of nineteen samples and sixteen features (phenolic compounds and tocopherols). PCA is an unsupervised chemometric method used for exploratory data analysis [42]. PCA selects the most important components to reduce data dimension and retain the variation of the data with the Principal Components (PCs) which are linear combinations of the variables of the dataset. The first PC explains the largest variance, the second PC presents the second largest variance, and so on [43]. HCA was also used to represent and visualize the classes of almonds, explore the similarities of the analyzed samples, and discover patterns among them [44]. A DT was developed in an attempt to discover patterns in the quantitative data and predict the geographical origin of the analyzed samples by assigning a numerical value. PCA and HCA were created in R using the MetaboAnalyst package [37]. The DT was created in Minitab 19 software (Minitab, PA, USA).

## 4. Conclusions

This work presents an innovative approach for assessing the bioactive content of almonds with the development of two RP-HPLC-UV methodologies for the determination of phenolic compounds and tocopherols, respectively. Nineteen samples of almonds originating from USA and Greece were analyzed, and gallic acid, ferulic acid, sinapic acid, caffeic acid, vanillic acid, p-coumaric acid, and rosmarinic acid were determined from the class of phenolic acids. Catechin, epicatechin, diosmin, quercetin, apigenin, luteolin, and kaempferol were determined from the class of flavonoids. Furthermore, from the group of tocopherols, α-tocopherol and the sum of (β + γ)-tocopherols were determined as well. The quantification results were further processed with chemometrics. PCA analysis quantitatively showed the distribution of the almonds on the score plot and the clear formation of two separate groups on the basis of their geographical origin (Greece or USA), with the first two PCs explaining the 66.7% of variance. An HCA dendrogram was built, as well, showing the clustering of two major groups according to the origin of production. Finally, a DT was developed for the prediction of the country of origin suggesting ferulic acid as a characteristic marker and proposing a concentration value of 1.54 μg/g.

The findings of this research have made progress towards the characterization of almonds that belong to the *Texas* variety, showing that the geographical origin affects the phenolic composition and tocopherol content, as well as showing that these bioactive constituents could be used for the authentication of almonds that are commercially available in the Greek market.

## Figures and Tables

**Table 1:** Phenolic compounds’ quantification results in Greek and American almonds (samples analyzed in triplicate, *n* = 3).

Phenolic Compounds	Greek Almonds		American Almonds	
	Concentration Range (μg/g)	Mean Value (±SD, μg/g)	Concentration Range (μg/g)	Mean Value (±SD, μg/g)
apigenin	4.65–8.65	7.04 ± 1.35	LOQ–3.21	1.77 ± 0.13
caffeic acid	1.18–1.85	1.53 ± 0.12	0.82–1.90	1.35 ± 0.14
catechin	11.7–27.8	21.3 ± 1.35	14.5–25.2	20.2 ± 1.11
diosmin	LOQ–29.0	3.91 ± 0.08	4.32–15.6	8.06 ± 0.54
epicatechin	3.21–6.01	5.13 ± 2.39	1.01–2.21	1.25 ± 0.08
ferulic acid	1.65–2.98	2.25 ± 0.23	LOQ–1.45	1.06 ± 0.09
gallic acid	LOQ–4.56	3.40 ± 0.18	1.14–1.81	1.51 ± 0.12
kaempferol	2.19–3.21	2.55 ± 0.66	LOQ–2.87	1.30 ± 0.19
luteolin	LOQ–0.65	0.59 ± 0.06	<LOQ	–
p-coumaric acid	<LOQ	–	<LOQ	–
quercetin	LOQ–0.68	0.53 ± 0.04	<LOQ	–
rosmarinic acid	1.03–1.84	1.34 ± 0.25	2.51–4.19	3.56 ± 0.75
sinapic acid	1.25–4.48	3.26 ± 0.85	1.02–3.65	2.18 ± 0.63
syringic acid	–	–	–	–
vanillic acid	1.37–4.25	3.31 ± 0.31	1.03–2.23	1.36 ± 1.22

**Table 2:** Tocopherols’ quantification results in Greek and American almonds (samples analyzed in triplicate, *n* = 3).

Tocopherol	Greek Almonds		American Almonds	
	Concentration Range (μg/g)	Mean Value (±SD, μg/g)	Concentration Range (μg/g)	Mean Value (±SD, μg/g)
α-tocopherol	502–802	643 ± 31	221–326	267 ± 18
sum of β- and γ-tocopherol	13.6–89.3	72.3 ± 5.7	61.2–81.2	68.2 ± 6.4

## Data Availability

Not applicable.

## Supplementary Materials

The following are available online, Figure S1: Characteristic chromatogram of an almond sample spiked at 5 μg/g. Figure S2: Characteristic chromatogram of a standard mixture of tocopherols at 10 μg/g. Figure S3: PCA biplot presenting the projection of the data set in PC1 and PC2. The red vectors show the influence to each PC (atoc: α-tocopherol; bctoc: β + γ-tocopherol). Figure S4: Prediction model performance characteristics. Table S1: Chromatographic retention times of the phenolic compounds determined in almonds. Table S2: Recoveries (%R) for the evaluation of repeatability. Table S3: Recoveries (%R) for the evaluation of intermediate precision. Table S4: Recoveries (%R) for the evaluation of repeatability. Table S5: Recoveries (%R) for the evaluation of repeatability. Table S6: Recoveries (%R) for the evaluation of intermediate precision. Table S7. Chromatographic retention times of the phenolic compounds determined in almonds.

## Sample Availability

Samples of the compounds are available from the authors.
