# Supplementary material for: A Rapid HPLC-UV Protocol Coupled to Chemometric Analysis for the Determination of the Major Phenolic Constituents and Tocopherol Content in Almonds and the Discrimination of the Geographical Origin
Source: Molecules. 2021 Sep 7;26(18):5433. doi: 10.3390/molecules26185433 (PMC8464707; doi:10.3390/molecules26185433)
Supplement: Supplementary file 1 [file molecules-26-05433-s001.zip › molecules-1365285-supplementary.pdf]

Supplementary Material

A rapid HPLC-UV protocol coupled to chemometric analysis for the determination of the major phenolic constituents and tocopherols content in almonds and the discrimination of the geographical origin

Natasa P. Kalogiouri, Petros Mitsikaris, Dimitris Klaoudatos, Athanasios Papadopoulos and Victoria F. Samanidou

Table S1. HPLC-UV analytical parameters for the determination of phenolic compounds.

Compound	Calibration equation	Linear range (μg/g)	r^2	LOD (μg/g)	LOQ (μg/g)
ferulic acid	y = 56.12x – 0.63	0.5 - 20	0.998	0.11	0.33
syringic acid	y = 44.80x – 2.11	0.5 - 20	0.999	0.14	0.42
epicatechin	y = 20.86x + 4.17	0.5 - 20	0.992	0.12	0.36
catechin	y = 18.13x + 6.12	2 - 20	0.997	0.42	1.26
p-coumaric acid	y = 81.81x + 4.11	1 - 20	0.998	0.31	0.93
sinapic acid	y = 23.92x + 2.44	1 - 20	0.993	0.28	0.84
kaempferol	y = 62.05x + 0.93	2 - 20	0.999	0.52	1.56
diosmin	y = 12.53 x – 0.35	2 - 20	0.995	0.60	1.80
quercetin	y = 389.16x – 16.42	0.5 - 20	0.998	0.15	0.45
vanillic acid	y = 63.62x + 4.37	1 - 20	0.992	0.31	0.93
epigallocatechin	y = 69.18x – 4.62	2 - 20	0.998	0.45	1.35
gallic acid	y = 33.72x + 0.44	0.5 - 20	0.992	0.05	0.15
caffeic acid	y = 137.18x – 7.57	0.5 - 20	0.996	0.25	0.75
apigenin	y = 22.32x + 0.81	2 - 20	0.996	0.44	1.32
luteolin	y = 37.76x + 1.51	2 - 20	0.997	0.52	1.56
rosmarinic acid	y = 65.53x + 0.90	0.5 - 20	0.998	0.08	0.24

LOD: limit of detection, LOQ: limit of quantitation

Table S2. Recoveries (%R) for the evaluation of repeatability.

Compound	Low Concentration (%R, n = 6)	%RSD	Medium Concentration (%R, n = 6)	%RSD	High Concentration (%R, n = 6)	%RSD
apigenin	92.2	5.1	96.3	1.7	87.9	3.2
caffeic acid	91.9	4.2	95.1	3.8	83.6	4.9
catechin	95.4	3.2	97.6	4.2	92.1	1.9
diosmin	95.2	4.5	88.2	4.8	92.6	5.5
epicatechin	94.2	4.1	88.1	5.6	86.7	4.3
ferulic acid	95.5	4.2	95.8	3.5	85.4	5.2
gallic acid	95.4	3.4	88.1	5.9	87.4	5.1
kaempferol	93.8	5.1	85.4	6.1	91.8	4.6
luteolin	92.8	4.6	87.4	4.4	90.2	4.3
p-coumaric acid	92.9	1.8	86.8	4.4	88.2	2.9
quercetin	93.1	4.6	95.9	5.4	95.5	4.2
rosmarinic acid	91.4	5.3	94.4	4.6	91.3	2.5
sinapic acid	95.4	3.3	87.3	5.4	85.6	5.1
syringic acid	90.4	1.6	87.5	5.3	83.1	2.6
vanillic acid	91.8	2.3	94.6	3.8	90.4	5.1

Table S3. Recoveries (%R) for the evaluation of intermediate precision.

**Compound**	**Low Concentration (%R, n = 3 x 3)**	**%RSD**	**Medium Concentration (%R, n = 3 x 3)**	**%RSD**	**High Concentration (%R, n = 3 x 3)**	**%RSD**
apigenin	102.2	6.4	98.8	6.3	95.6	6.5
caffeic acid	97.8	6.2	97.4	7.4	94.5	5.2
catechin	95.4	3.2	96.4	5.5	93.7	5.2
diosmin	102.2	7.5	90.2	8.3	94.4	7.2
epicatechin	98.2	7.4	99.1	6.6	94.4	6.6
ferulic acid	100.4	7.2	97.2	4.1	95.4	7.8
gallic acid	99.1	8.5	95.1	6.1	91.2	6.2
kaempferol	101.6	10.1	92.4	7.2	95.4	6.4
luteolin	97.6	5.6	92.2	8.2	93.8	6.4
p-coumaric acid	101.2	8.8	98.5	3.7	95.4	4.1
quercetin	98.1	7.5	92.3	5.9	96.7	6.4
rosmarinic acid	103.4	7.7	97.2	5.5	96.7	3.3
sinapic acid	98.4	9.2	93.3	6.5	93.6	5.6
syringic acid	95.4	6.6	100.5	6.2	92.8	6.4
vanillic acid	97.5	8.6	95.5	10.5	93.2	8.1

Table S4. HPLC-UV analytical parameters for the determination of tocopherols.

**Compound**	**Calibration equation**	**Linear range (μg/g)**	**r^2**	**LOD (μg/g)**	**LOQ (μg/g)**
α-tocopherol	y = 6.44x – 1.65	5 – 50	0.995	0.33	0.99
β+γ-tocopherol	y = 7.21x + 0.10	5 – 50	0.996	0.12	0.36
δ-tocopherol	y = 6.21x + 0.59	5 - 50	0.998	0.22	0.66

LOD: limit of detection, LOQ: limit of quantitation

Table S5. Recoveries (%R) for the evaluation of repeatability.

Compound	Low Concentration (%R, n = 6)	%RSD	Medium Concentration (%R, n = 6)	%RSD	High Concentration (%R, n = 6)	%RSD
α-tocopherol	99.2	3.1	96.5	4.4	95.1	5.5
β+γ-tocopherol	100.4	2.6	97.2	3.9	96.1	4.2
δ-tocopherol	98.7	4.2	95.4	3.3	95.6	4.9

Table S6. Recoveries (%R) for the evaluation of intermediate precision.

Compound	Low Concentration (%R, n = 3 x 3)	%RSD	Medium Concentration (%R, n = 3 x 3)	%RSD	High Concentration (%R, n = 3 x 3)	%RSD
α-tocopherol	95.1	5.7	94.5	5.9	94.7	6.6
β+γ-tocopherol	96.8	7.2	94.2	7.7	95.3	7.6
δ-tocopherol	94.6.7	6.2	93.2	8.1	96.2	8.1

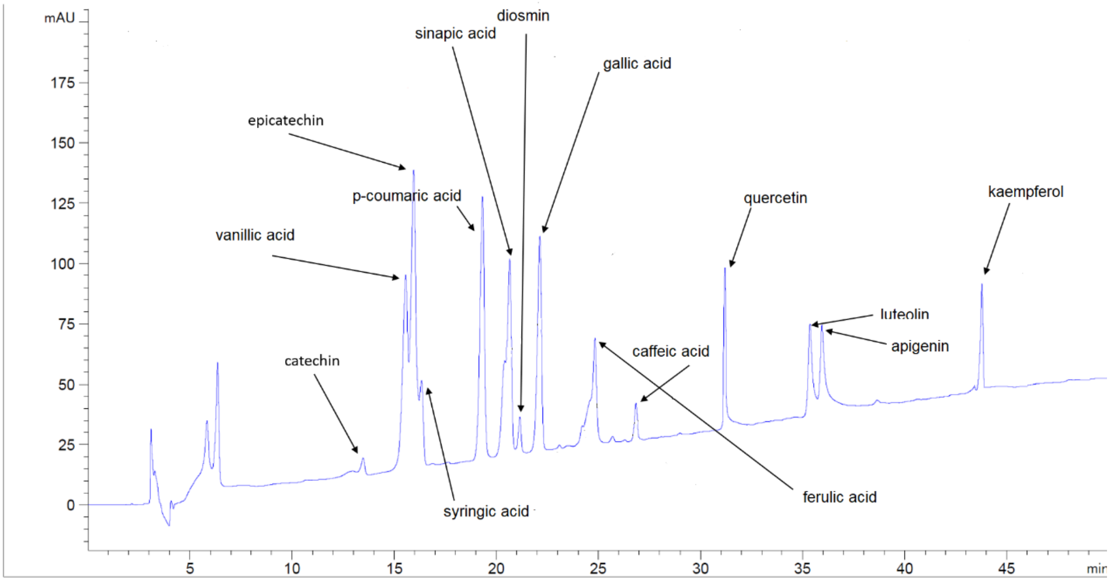

Figure S1. Characteristic chromatogram of an almond sample spiked at 5µg/g and monitored at 280 nm.

Table S7. Chromatographic retention times of the phenolic compounds determined in almonds.

Phenolic compound	Rt (min)
catechin	13.9
vanillic acid	15.2
epicatechin	16.1
syringic acid	16.8
p-coumaric acid	18.9
sinapic acid	20.8
diosmin	21.1
gallic acid	22.3
ferulic acid	24.9
caffeic acid	27.1
quercetin	31.3
luteolin	35.2
apigenin	36.5
kaempferol	43.5

Rt: retention time

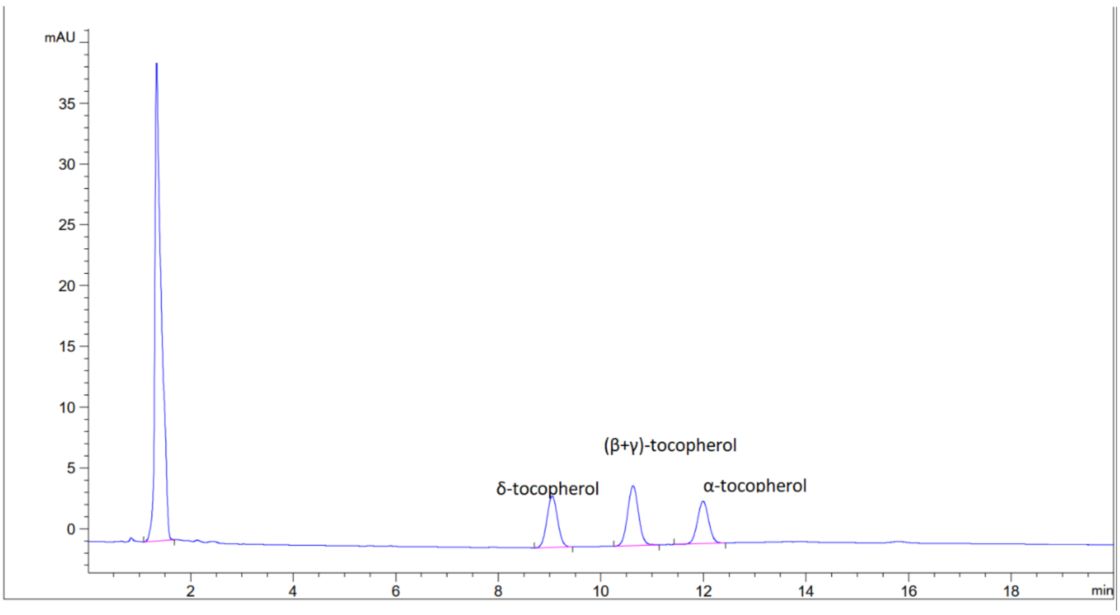

Figure S2. Characteristic chromatogram of a standard mixture of tocopherols at 10 μg/g and monitored at 295 nm.

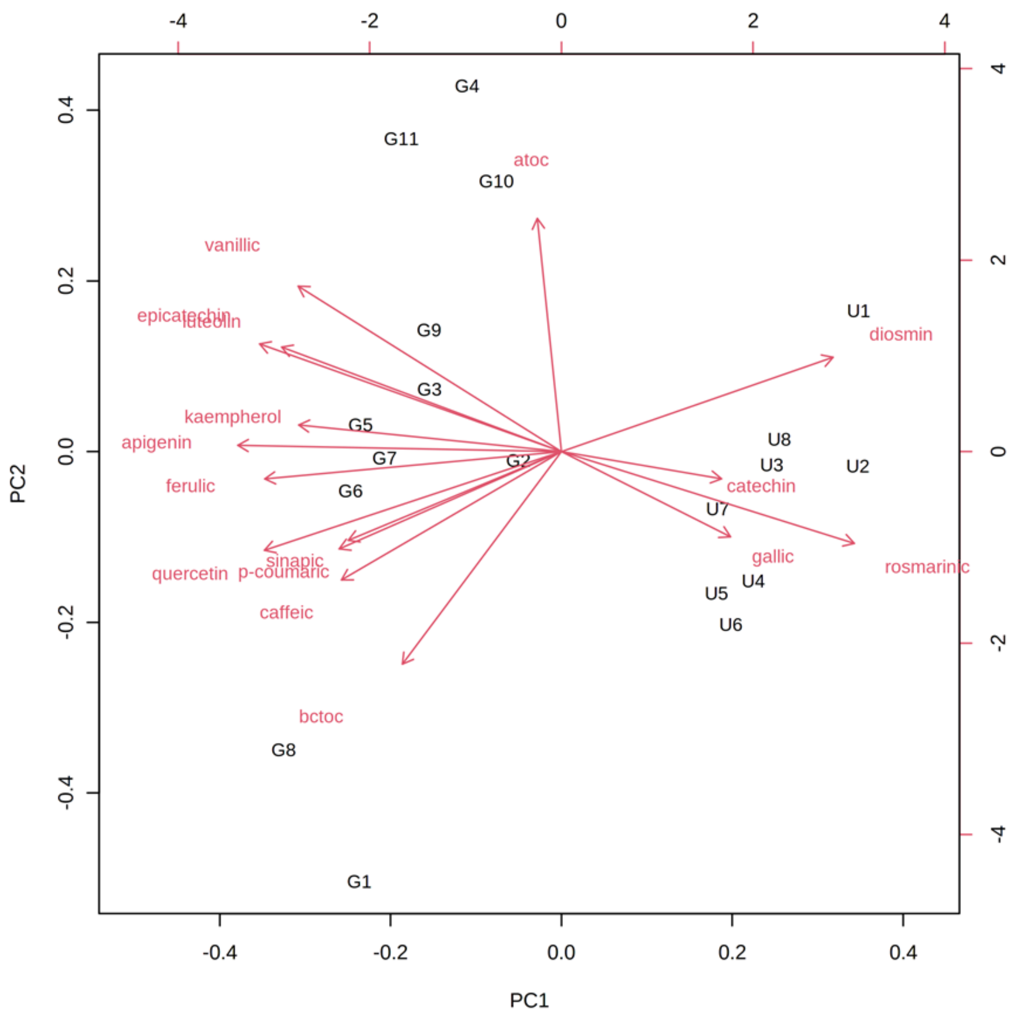

Figure S3. PCA biplot presenting the projection of the data set in PC1 and PC2. The red vectors show the influence on each PC (atoc: α-tocopherol; bctoc: β+γ-tocopherol).

Model Summary

Total predictors	16
Important predictors	11
Number of terminal nodes	2
Minimum terminal node size	3

Statistics	Training	Test
Average -loglikelihood	0,0000	0,0000
Area under ROC curve	1,0000	1,0000
95% CI	(0; 1)	(0; 1)
Lift	4,0000	1,6000
Misclassification cost	0,0000	0,0000

Figure S4. Prediction model performance characteristics.
